# Population Genetic Analysis of *Plasmodium falciparum* Parasites Using a Customized Illumina GoldenGate Genotyping Assay

**DOI:** 10.1371/journal.pone.0020251

**Published:** 2011-06-06

**Authors:** Susana Campino, Sarah Auburn, Katja Kivinen, Issaka Zongo, Jean-Bosco Ouedraogo, Valentina Mangano, Abdoulaye Djimde, Ogobara K. Doumbo, Steven M. Kiara, Alexis Nzila, Steffen Borrmann, Kevin Marsh, Pascal Michon, Ivo Mueller, Peter Siba, Hongying Jiang, Xin-Zhuan Su, Chanaki Amaratunga, Duong Socheat, Rick M. Fairhurst, Mallika Imwong, Timothy Anderson, François Nosten, Nicholas J. White, Rhian Gwilliam, Panos Deloukas, Bronwyn MacInnis, Christopher I. Newbold, Kirk Rockett, Taane G. Clark, Dominic P. Kwiatkowski

**Affiliations:** 1 Wellcome Trust Sanger Institute, Hinxton, United Kingdom; 2 Global Health Division, Menzies School of Health Research, Charles Darwin University, Darwin Northern Territory, Australia; 3 Institut de Recherche en Sciences de la Santé, Direction Régionale de l'Ouést, Bobo-Dioulasso, Burkina Faso; 4 Section of Parasitology, Department of Public Health Sciences, University of Rome La Sapienza, Rome, Italy; 5 Malaria Research and Training Centre, Faculty of Medicine, Pharmacy, and Odontostomatology, University of Bamako, Bamako, Mali; 6 KEMRI/Wellcome Trust Research Program, Kilifi, Kenya; 7 Papua New Guinea Institute of Medical Research, Goroka, Papua New Guinea; 8 Faculty of Health Sciences, Divine Word University, Madang, Papua New Guinea; 9 Laboratory of Malaria and Vector Research, National Institute of Allergy and Infectious Diseases, National Institutes of Health, Bethesda, Maryland, United States of America; 10 National Center for Parasitology, Entomology and Malaria Control, Phnom Penh, Cambodia; 11 Departments of Clinical Tropical Medicine and of Molecular Tropical Medicine and Genetics, Faculty of Tropical Medicine, Mahidol University, Bangkok, Thailand; 12 Southwest Foundation for Biomedical Research, San Antonio, Texas, United States of America; 13 Shoklo Malaria Research Unit, Mae Sot, Thailand; 14 Mahidol-Oxford University Research Unit, Faculty of Tropical Medicine, Bangkok, Thailand; 15 The Weatherall Institute of Molecular Medicine, John Radcliffe Hospital, Oxford, United Kingdom; 16 Wellcome Trust Centre for Human Genetics, University of Oxford, Oxford, United Kingdom; 17 London School of Hygiene & Tropical Medicine, London, United Kingdom; University of Edinburgh, United Kingdom

## Abstract

The diversity in the *Plasmodium falciparum* genome can be used to explore parasite population dynamics, with practical applications to malaria control. The ability to identify the geographic origin and trace the migratory patterns of parasites with clinically important phenotypes such as drug resistance is particularly relevant. With increasing single-nucleotide polymorphism (SNP) discovery from ongoing *Plasmodium* genome sequencing projects, a demand for high SNP and sample throughput genotyping platforms for large-scale population genetic studies is required. Low parasitaemias and multiple clone infections present a number of challenges to genotyping *P. falciparum*. We addressed some of these issues using a custom 384-SNP Illumina GoldenGate assay on *P. falciparum* DNA from laboratory clones (long-term cultured adapted parasite clones), short-term cultured parasite isolates and clinical (non-cultured isolates) samples from East and West Africa, Southeast Asia and Oceania. Eighty percent of the SNPs (n = 306) produced reliable genotype calls on samples containing as little as 2 ng of total genomic DNA and on whole genome amplified DNA. Analysis of artificial mixtures of laboratory clones demonstrated high genotype calling specificity and moderate sensitivity to call minor frequency alleles. Clear resolution of geographically distinct populations was demonstrated using Principal Components Analysis (PCA), and global patterns of population genetic diversity were consistent with previous reports. These results validate the utility of the platform in performing population genetic studies of *P. falciparum*.

## Introduction

*Plasmodium falciparum* continues to impose a substantial public health burden across the globe, causing an estimated 500 million clinical cases and 1–2 million deaths each year [1]. The lack of an effective antimalarial vaccine and the emergence and spread of parasite resistance to affordable antimalarial drugs such as chloroquine and sulfadoxine-pyrimethamine, have greatly contributed to the public health burden of malaria [2]. More worryingly, reports are appearing of evolving resistance to artemisinin combination therapies, currently the frontline treatment throughout the world. [3]. Effective measures to control malaria and reduce parasite transmission in the face of drug resistance are urgently needed.

Subsets of genetic polymorphisms can be used to explore the dynamics of parasites with important phenotypes such as drug resistance, with practical applications to malaria control and elimination. Key to the success of malaria control strategies is an understanding of the parasite genetic diversity and dynamics/exchange of gene flow between human populations. With this knowledge, effective spatial and temporal boundaries for intervention can be implemented. The ability to identify the geographic origin and monitor the migration patterns of clinically important parasite genetic traits should greatly facilitate control efforts, with important applications for drug resistance surveillance. With sufficient genetic information, we may be able to use the spectrum of genetic variation, or in a more simplified form, a “molecular barcode”, to elucidate the geographic origin of an infection. Molecular barcodes have already been used successfully by Daniels and colleagues [4] to distinguish parasite clones from one another.

With ongoing whole-genome *Plasmodium* sequencing efforts providing a valuable resource of SNPs (http://www.sanger.ac.uk/research/projects/malariaprogramme-kwiatkowski/), and enhancing the feasibility of large scale genotype-based population genetic studies of the parasite, a demand for effective high sample and SNP throughput genotyping platforms is anticipated. However, genotyping *P. falciparum* samples presents a number of challenges including limited DNA quantity, an abundance of contaminating human DNA, extensive polymorphism that may occur in primer binding sites, and multiple clone infections in which multiple parasite clones may be present at any number and ratio.

To address some of these limitations, we developed a customized Illumina GoldenGate 384-SNP genotyping assay. The GoldenGate platform was chosen for the study owing to the moderately high sample (96 samples in parallel) and SNP (up to 1536 loci) throughput, and the custom design feature, which enables users to tailor the SNP genotyping assay to meet the specific requirements of their study. Using *P. falciparum* clones and isolates from East and West Africa, Southeast Asia and Oceania, we measured the specificity and sensitivity of the GoldenGate platform on various preparations of *P. falciparum* DNA. In addition, we characterise the genetic structure of parasite populations and assess the potential of molecular barcoding approaches to identify the likely geographic origin of an infection.

## Materials and Methods

### Parasite samples and DNA preparation

*P falciparum* DNA was obtained from either i) long-term cultured parasite clones (laboratory parasite 3D7, HB3 and IT), ii) short-term culture parasite isolates obtained from patients with malaria (in Kenya, Cambodia and Thailand), and (iii) non-cultured parasite isolates obtained directly from patient's blood (malaria clinical samples collected in Mali, Burkina Faso and Papua New Guinea (PNG) (Table S1). Laboratory clones and short-term parasite cultures were grown *in vitro* as previously described [5]. All samples were collected with written informed consent from adult patient or a parent or guardian of paediatric patients. The projects were approved by all the relevant ethical committees (Comite d'Ethique de la Faculté de Médecine de Pharmacie et d'Odontostomatologie, Bamako, Mali, Comite d'Ethique Institutionnel du Centre Muraz, Bobo- Dioulasso, Burkina Faso, Institutional Review Board (IRB) of National Institute of Allergy and Infectious Diseases, United States, Cambodian National Ethics Committee for Health Research, IMR Institutional Review Board, Madanga, Medical Research Advisory Committee of Papua-New-Guinea, Ethics Committee of the Faculty of Tropical Medicine at Mahidol University , Bangkok, Thailand, KEMRI scientific, ethical and export license approval, Oxford Tropical Research Ethical Committee (OxTREC) ethical approval and Heidelberg ethical approval for Kenyan samples).

Prior to DNA extraction, all blood samples were subject to white blood cell depletion to reduce human DNA contamination using a combination of lymphoprep density gradient centrifugation (Axis-Shield) followed by Plasmodipur filtration (Euro-Diagnostica), as described in (Auburn *et al.*, submitted). Genomic DNA was extracted from infected red blood cell pellets using the QIAamp DNA Blood Midi Kit according to the manufacturer's protocol (QIAGEN). Whole genome amplification was undertaken on DNA template (5–10 ng of total DNA) using Genomiphi's Multiple Displacement Amplification kit as per protocol (Amersham Biosciences). Human DNA extracted from an EBV-transformed B cell line obtained from the Centre d'Etude du Polymorphisme Humain collection (Coriell Institute for Medical Research) was used as a control and in preparations of human-spiked *Plasmodium* samples for the assessment of the effect of human DNA presence on *Plasmodium* genotyping efficacy. To address the quantities of human and *Plasmodium* DNA in each sample, quantitative real time PCR (QRT-PCR) analysis using human and parasite-specific primers was undertaken using the Applied Biosystems StepOne RT-PCR system (Auburn *et al.*, submitted).

### SNP selection and genotyping

An Illumina 384-SNP custom GoldenGate assay was used to genotype *P. falciparum* samples. SNPs were identified in PlasmoDB version 4.4 (www.plasmoDB.org) [6] using the following criteria: (a) polymorphic between 3 *P. falciparum* laboratory clones (3D7, possible African origin, HB3 from Honduras, IT from Brasil) with at least 1 observation of each allele, (b) Illumina assay design score >1 (scores are determined by the Illumina Assay Design Tool and minimum recommendations are based on Illumina internal testing) (c) uniform spacing across the *P.falciparum* genome. The final set of 384 SNPs consisted of 31 well-known candidate gene SNPs and 353 SNPs chosen to achieve on average 1 marker per 60 kb (Table S2). The reference sequence genome of the 3D7 parasite clone [7] was used for assay design. Genotyping was performed using the manufactures protocol on 96-well format Sentrix arrays [8]. This system uses a high-density BeadArray technology in combination with an allele-specific extension, adapter ligation and amplification assay protocol. Briefly, biotinylated DNA was immobilized on paramagnetic beads and the pooled SNP-specific oligonucleotides were annealed on the DNA. Oligonucleotides that hybridized were then extended and ligated to generate DNA templates which were amplified using universal fluorescently-labelled primers. Single-stranded PCR products were hybridized to a Sentrix® Array Matrix. Arrays were imaged using a BeadArray Reader Scanner. All samples were genotyped at least in duplicate in the same array/plate. A number of samples were repeated in different plates. Laboratory clones were genotyped using a range of DNA quantities (0.25–250 ng of total DNA). A minimum of 2–5 ng of total DNA was used to genotype the remaining samples.

### Assay performance

**Figure 1:**
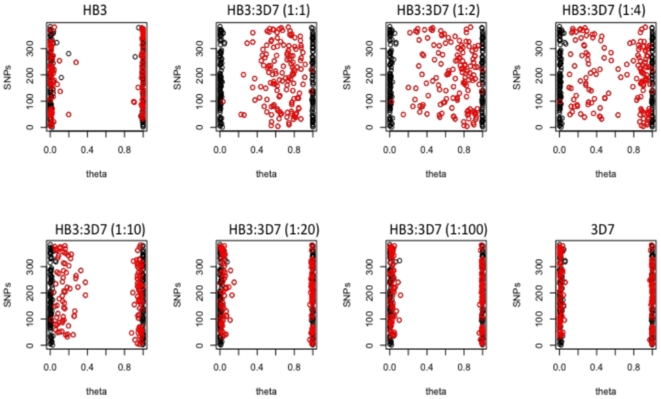
Distribution of theta values (proxy to genotype call) at 319 “reliable” SNPs in preparations from the laboratory clones 3D7 and HB3, and in artificial mixtures of the two clones. Mixing proportions of the clones (HB3:3D7) are indicated in brackets. Red dots represent the SNPs that should have heterozygous genotypes in the artificial mixes (according to the genotypes from the haploid pure samples), black dots represent the SNPs for which both samples have the same genotype. Theta values approximating 0.5 correspond to having both alleles approximating 50% frequency. Values approximating 0 and 1 correspond to homozygous calls for allele a or b. In the pure clonal 3D7 and HB3 samples, the majority of genotype calls exhibit theta values <0.1 and >0.9.

Illumina's Bead Studio software converts allele intensities for each SNP into polar coordinate-based variables to call genotypes: allelic intensity ratios (theta) that range from 0 to 1 and intensity values (R) with values from 0 onwards. Theta values approximating 0 and 1 reflect different alleles and, thus, theta is used to score genotypes as heterozygous or homozygous for each allele [9]. R values represent overall SNP intensity, whereby values <0.1 are considered poor intensity [9]. The genotype calling strategy was based on the distribution of theta values in pure and artificial mixes of laboratory clones, where the expected genotype call is known (see Figure 1). Correlations between the theta values were estimated to assess the degree of concordance between replicates (Pearson's correlation coefficient). Additionally, genotype concordance was assessed by comparing genotype calls and by comparing replicate standard deviations (concordance of theta values was considered for standard deviation <0.05). SNPs with R<0.1 and/or no correlation between replicates were considered failures and removed from further analysis.

### Population genetic analysis

Expected heterozygosity (*H_E_*) at each locus was calculated as *H_E_* = n/(n-1)](2*pq*), where n is the number of infections sampled, *p* is the frequency of allele 1 and *q* is the frequency of allele 2. The formula [n/(n-1)] was included to enable adjustment for sample size. At the population level, *H_E_* was averaged across all loci [10].

Principal component analysis (PCA) was performed by calculating the eigenvalues and their vectors (PCs) of a matrix consisting of average number of pairwise genotype or theta differences between each isolate. The proportion of total variation explained by each principal component was estimated using eigenvalues. The genetic differentiation between populations was assessed using pair-wise measures of Wright's Fixation index (F_ST_), using both theta values and genotype calls [11]. Calculations were only undertaken where the sample size of both populations was >15. Sets of informative SNPs for assessment of population divergence were constructed by ranking F_ST_ statistics (Cockerham and Weir) [10] calculated at each SNP position.

## Results

### Assay performance on laboratory clones: 3D7, HB3, IT

Validation of the 384-SNP assay was undertaken using genomic DNA from the 3D7 parasite that was used for assay design. A set of reliable SNPs was identified using the following criteria: (a) allelic concordance with the reference sequence [7], (b) genotype intensity (R>0.1), (c) genotype concordance between replicates, and d) homozygote genotype status (under the assumption of 3D7 clonality). As a result, 319 reliable SNPs (83.1% of total) were selected for subsequent analyses (Table S2). A high degree of correlation (r^2^>0.99) was observed for both inter- and intra-plate replicates for this set of SNPs, demonstrating overall low experimental variability on the GoldenGate platform (Table 1). The performance of the platform was further tested across the reliable SNPs using DNA from HB3 and IT laboratory clones. More than 95% of the 319 loci analysed performed well, as demonstrated by the genotype intensity signals and genotype concordance between replicates (Table 1). Approximately 25% of these SNPs were monomorphic between the 3 laboratory clones (Table S2).

**Table 1:** Assessment of assay performance using laboratory clones 3D7, IT and HB3.

Sample	Genomic DNA (ng)	n° SNPs analysed^a^	Intensity (R>0.1)	Filtration A^b^	Filtration B^c^	Correlation between replicates^d^
			n° SNPs	n° SNPs	n° SNPs	
3D7	**250**	**384**	**343**	**339**	**319**	**1.000**
	25	319	319	319	319	1.000
	2.5	319	319	319	317	0.999
	0.25	319	318	313	312	0.999
HB3	250	319	305	315	304	0.999
	25	319	303	305	301	0.998
	2.5	319	303	302	300	0.999
	0.25	319	305	244	237	0.998
IT	250	319	305	307	303	0.999
	25	319	305	311	304	0.999
	2.5	319	304	306	300	0.995
	0.25	319	307	294	290	0.999

a 319 SNP set was defined from assessments of genotype concordance in 250 ng replicates of the 3D7 reference strain.

b Filtration criteria A: SNPs with genotype concordance between replicates >0.95.

c Filtration criteria B: SNPs with R>0.1 and with genotype concordance between replicates >0.95.

d Mean correlation between replicates.

### Genotyping efficacy with varying DNA quantity and preparation

The ability of the assay to perform at lower DNA quantities than recommended by Illumina (total of 250 ng of DNA) was investigated by assessing the intensity and genotype concordance between 250 ng, 25 ng, 2.5 ng and 0.25 ng templates of the clones 3D7, HB3 and IT. For all three parasite clones, the performance of the 25 ng and 2.5 ng templates was similar to the 250 ng template (Table 1). Although genotype concordance was lower at 0.25 ng, the overall concordance rate was still moderately high (97.8%, 77.9% and 95.7% of SNPs for 3D7, HB3 and IT, respectively).

The influence of human DNA on *P. falciparum* genotyping accuracy was also assessed by comparison of the genotype calls between samples containing only parasite DNA with samples containing a mixture of parasite and human DNA at different concentrations (Table S3). Genotype calls were consistent between the samples containing only parasite DNA and the mixed samples containing only 0.01% or more of parasite DNA (genotype correlation: r^2^>0.96).

Genotyping efficacy was also assessed on *P. falciparum* samples subject to whole genome amplification (WGA) using the multiple displacement amplification (MDA) method [12], [13]. WGA products were tested on templates from 3D7, mixtures of 3D7 and human DNA (1∶1 ratio), and two clinical Malian samples (non-cultured) (Table S4). QRT-PCR on the pre- and post-amplification products confirmed that the human DNA had not impeded amplification of the parasite DNA during the MDA process. More than 99.4% of the 319 reliable SNPs passed the inclusion criteria on both the pure 3D7 and in the human:3D7 mixtures. Furthermore, ∼100% genotype call concordance was observed both between replicates and between the pure 3D7 and the human:3D7 mixtures. Reliable and reproducible results were also obtained in comparisons of genomic DNA with the WGA samples prepared from the two Malian clinical isolates (Table S4).

### Genotyping efficacy on clinical and short-term cultured samples

Given that SNP selection and primer design were based on the SNP information available in PlasmoDB version 4.4, it is possible that unidentified SNPs might reduce the genotyping efficacy at a number of loci, particularly in sample populations that were not well represented in this version of the database. The genotyping efficacy at the 319 reliable SNPs was assessed in 143 short-term or clinical (non-cultured) samples sourced from Burkina Faso, Mali, Kenya, Cambodia, Thailand and Papua New Guinea. As summarized in Table 2, on average, 95.5% of the SNPs performed well across all samples. Thirteen SNPs exhibited low intensity calls (R<0.1) in more than 80% of samples and were excluded from further analysis on this sample set, leaving a set of 306 “globally reliable SNPs” (Table S2). The minor allele frequency (MAF) for each SNP is presented in Table S2. On this set of 143 samples, 267 SNPs displayed a MAF ≥5%.

**Table 2:** Assessment of assay performance using *P. falciparum* samples obtained from patients with malaria (short-term and non-cultured samples).

		Intensity (R>0.1)	Filtration A^a^	Filtration B^b^	Correlation between replicates^c^
Origin	No. samples	n° SNPs	n° SNPs	n° SNPs	
PNG	23	310	312	309	0.997
Thailand	19	313	311	306	0.998
Cambodia	22	299	310	295	0.996
Kenya	19	310	305	304	0.998
Burkina Faso	37	308	314	307	0.999
Mali	23	311	313	307	0.999

Samples assessed on 319 “reliable” SNPs.

a Filtration criteria A: SNPs with genotype concordance between replicates >0.95.

b Filtration criteria B: SNPs with R>0.1 and with genotype concordance between replicates >0.95.

c Mean correlation between replicates.

Clinical malaria samples obtained directly from patient's blood may have multiple genetically distinct *P. falciparum* clones. The presence of heterozygous genotypes is indicative of the presence of more than one clone (haploid genome) in a single infection (multiclonality). Examples of the genotype profiles (as theta scores) of 2 clinical samples (non-cultured) described in this study are presented in Figure S1. In general, the clinical samples from Africa exhibited a greater number of heterozygous calls than the clinical samples from PNG. This observation complies with the results from a large-scale whole genome sequencing dataset (Manske *et al*., under review). Whole genome sequence data generated on the Illumina Genome Analyzer platform was available for 105 of the samples described in this study. Thus, it was possible to assess the reliability of the GoldenGate genotype calls, by comparison with the Genome Analyzer calls at 106 of the globally reliable SNPs. . The full set of globally reliable SNPs could not be compared owing to regions of low sequence coverage. At the 106 loci, an average genotype concordance of 97.8% (including both homozygous and heterozygous genotypes) was observed between the two datasets (Manske *et al*., under review).

### Genotype calling sensitivity in mixtures of laboratory clones

In contrast to clonal laboratory parasites [14], clinical malaria infections may carry multiple clones present in any number and at any ratio. In order to assess the sensitivity of the GoldenGate platform to detect the presence of multiple clones (i.e. minor alleles), mixtures of DNA from the laboratory clones 3D7 and HB3 were prepared at known ratios (1∶1, 1∶2, 1∶4, 1∶10, 1∶20, 1∶50, and 1∶100). Figure 1 presents a selection of plots illustrating the genotype profiles (theta values) for the pure clones and for the mixtures. In the pure HB3 and 3D7 samples, the large majority of SNPs (>99%) exhibit theta scores <0.1 and >0.9 (homozygous genotypes, as clones are haploid). Thus, a theta value between 0.1–0.9 was employed for assignment of heterozygous status (in this case, indicating the presence of more than one haploid clone). As the genotype calls for each individual clone were known, it was possible to determine which *loci* should exhibit heterozygous genotypes in the mixtures. At ratios of 1∶1, only 5 SNPs expected to exhibit heterozygous genotypes could not be distinguished from homozygous loci. All other expected heterozygous loci were clearly distinguishable from the homozygous loci, and exhibited theta values between 0.1–0.9. At ratios of 1∶2 and 1∶4, it was still possible to discriminate 84.7% and 71.5% of the expected heterozygous SNPs (theta between 0.1–0.9). At ratios of 1∶10, discrimination had decreased to 34.7%. At lower ratios, the assay clearly tended toward the genotype calls of the predominant parasite and it was not possible to discriminate different clones within the mixture.

### Population genetics: population diversity, differentiation and geographic positioning

Using the data from the 143 clinical and short-term cultured samples at the 306 globally reliable SNPs (Table S2), we assessed several population genetic parameters including the genetic diversity within each population, inter-population differentiation, and the ability to assign geographic origins to samples on the basis of their Principal Component Analysis (PCA) clustering pattern. Analysis was performed using both homozygous and heterozygous calls. In general, the large majority of loci displayed homozygous calls. On average, <0.6% of the calls in the samples from PNG and <8% of the calls in the samples from Africa were heterozygous. Indeed, where heterozygous calls were observed, as illustrated in Figure S1, the majority of these displayed a theta score close to 0 or 1, indicative of a predominant allele. This minor allele frequency spectrum has also been observed in Illumina Genome Analyzer sequence data generated on clinical samples from Africa (Manske *et al*., under review).

The level of genetic diversity in each population was assessed using a measure of the expected heterozygosity with adjustment for sample size (Table 3). Global patterns of *P. falciparum* diversity were consistent with previous reports [15], [16], [17], [18], with higher levels of diversity in the three African populations, Kenya, Mali and Burkina Faso (*H_E_* = 0.23, 0.24, and 0.24, respectively), than in the Thailand, Cambodia and Papua New Guinea samples (*H_E_* = 0.17, 0.18, and 0.18, respectively). This would fit with the view that *P. falciparum* originated in Africa, and that some of the ancestral diversity has been lost as it has spread to other parts of the world; an alternative explanation might be that the level of diversity is related to historical levels of transmission intensity, which have tended to be higher in Africa than in Southeast Asia.

**Table 3:** Population genetic characterisation of 143 *P. falciparum* samples obtained from patients with malaria (short-term and non-cultured samples) using 306 “globally reliable” SNPs.

	Wright's F_ST_					Expected Heterozygosity (s.d.)^a^
Country	Thailand	Cambodia	Kenya	Burkina Faso	Mali	
PNG	0.253	0.243	0.275	0.389	0.391	0.18 (0.19)
Thailand		0.089	0.329	0.463	0.456	0.17 (0.21)
Cambodia			0.326	0.457	0.454	0.18 (0.20)
Kenya				0.088	0.092	0.23 (0.21)
Burkina Faso					0.011	0.24 (0.21)
Mali						0.24 (0.20)

a Expected heterozygosity at each locus was calculated as *H_E_* = [*n*/(*n* - 1)][2*pq*]: see Materials and Methods.

Pair-wise estimates of the differentiation between the parasite populations were calculated using Wright's Fixation index (F_ST_) [11]. A summary of these results is presented in Table 3. Low levels of differentiation were observed within continental boundaries (all F_ST_<0.1), particularly between Mali and Burkina Faso (F_ST_<0.011). High levels of differentiation were observed between Papua New Guinea and all other populations, with a trend of increasing differentiation by distance (PNG F_ST_ comparisons: Cambodia = 0.243, Thailand = 0.253, Kenya = 0.275, Burkina Faso = 0.389, and Mali = 0.391). A similar trend of increasing differentiation by distance was observed between the Asian and African populations. High levels of differentiation were observed between Kenya and each of Cambodia (F_ST_ = 0.326) and Thailand (F_ST_ = 0.329), but the highest levels of differentiation were observed between the Asian and West African populations (F_ST_ range 0.454–0.463). As with the heterozygosity patterns, these global patterns of population differentiation are consistent with previous reports for *P. falciparum* [19], [20].

**Figure 2:**
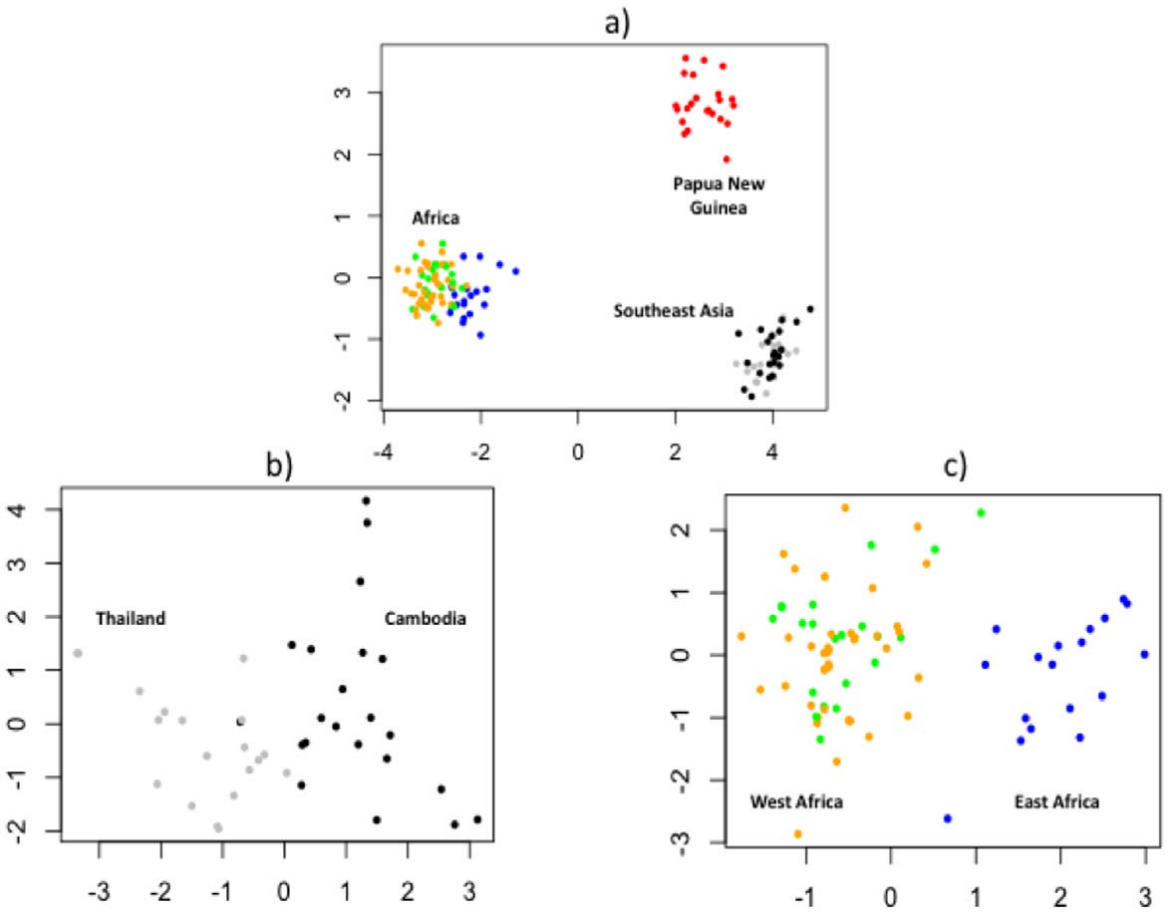
Principal Components Analysis plots (x axis represents PC1, and y axis PC2) on the 143 clinical (non-cultured) and short-termed cultured samples using the 306 “globally reliable” SNPs. Green = Mali Orange = Burkina Faso, Blue = Kenya, Black = Cambodia, Grey = Thailand, Red = Papua New Guinea. Clear continental differentiation of samples is observed (a). Within continental boundaries, moderate resolution of Thailand from Cambodia (b), and East Africa from West Africa (c) is observed.

Principal Components Analysis (PCA) was used to assess the ability to assign the geographic origin of a given sample on the basis of its clustering pattern and underlying molecular barcode (Figure 2). Using the 306 globally reliable SNPs, distinct clustering of continental populations was observed (Africa, Southeast Asia and Oceania), with moderate distinction of intra-continental geographic boundaries (Thailand versus Cambodia, and West Africa versus East Africa). Despite the political assignment of Mali and Burkina Faso as separate populations, levels of gene flow between these populations appeared to be high, with no distinct clustering. Thus, it was not possible to unambiguously assign samples to either country. By calculating the F_ST_ statistics [10] at each SNP for different pair-wise population comparisons, it was possible to rank the polymorphisms by their ability to differentiate populations. Several alleles have reached fixation (or very high allele frequencies) in certain populations enabling the identification of a set of SNPs that can effectively discriminate samples from different geographic locations, for example, between continents or countries. In doing so, it was possible to ascertain a minimum number of informative SNPs (i.e., molecular barcode) that would clearly differentiate sample populations. Using this approach, a 9-SNP molecular barcode was identified which could resolve samples at the continental level, a 6-SNP barcode with modest resolution of Thai and Cambodian samples, and a 4-SNP barcode with modest resolution of East and West African samples (Figure S2 and Table S2). Using a 13-SNP barcode, low resolution of Mali and Burkina Faso was possible, but more informative markers are required to improve this resolution (increasing the number of SNPs did not improve separation). As the informative SNPs were selected on the basis of allele frequencies at or close to fixation within a population, low levels of heterozygous calls are generally observed within populations at these SNP loci. Indeed, the informative SNP data sets did not change when excluding data with heterozygous genotyping calls. The sets of informative SNPs are expected to change with further information from larger sample sets from broader geographic regions. Ongoing whole genome sequencing projects should facilitate identification of more informative markers for high-resolution molecular barcodes.

## Discussion

To successfully implement and monitor malaria control and elimination strategies, we need a greater understanding of the dynamics of parasite gene flow between populations and effective and rapid tools for tracing important parasite genetic traits such as anti-malarial drug resistance. Microsatellite-based approaches are common-place for elucidating the population genetic structure of parasite populations [19], [20], and various methods including *msp* typing [21] and a 24-SNP TaqMan assay [4] have been described for genetically distinguish *P. falciparum* parasites.

Rapidly accumulating genome-wide *P. falciparum* SNP datasets (Manske *et al*., under review) [20], [22], [23], [24] should greatly enhance the feasibility for large-scale genotype-based population genetic studies of the parasite. However, genotyping *P. falciparum* samples is challenging owing to features such as limitations in the quantity of parasite DNA, contamination with human DNA from white blood cells, and the presence of extensive polymorphism, including multiple clone infections.

We assessed the utility of the high-throughput Illumina GoldenGate platform to genotype various preparations of *P. falciparum* DNA, and to apply these data to population genetic studies of the parasite. The Illumina BeadXpress platform, which primarily uses the Golden Gate SNP genotyping assay but genotypes lower number of SNPs, has been previously successful on genotyping 96 SNPs in *P. falciparum* [25]. However, the ability of this method to quantify alleles present in multiple clone infections and the robustness of the assay in the face of human DNA contamination and limiting starting DNA template had not been thoroughly explored.

Using a 384-SNP custom-design GoldenGate assay, under strict criteria for genotype intensity (R>0.1) and concordance between sample replicates (r^2^>0.95), 84% (306) of SNPs demonstrated reliable genotyping performance on both clinical and cultured samples from a range of geographic locations, including samples with abundant human contamination and with low quantities of genomic or whole genome amplified (WGA) DNA.

Multiple clone infections present a challenge to parasite genetic studies owing to sensitivity of detection of minor frequency variants and potential ambiguity in haplotype reconstruction. Accurate haplotype reconstruction is important for certain genetic metrics which require an understanding of genetic phase, such as *linkage disequilibrium* (LD). Patterns of LD underlie methods to detect signatures of recent positive selection, and candidate loci underlying traits such as antimalarial drug resistance [26]. Haplotypes can also facilitate the identification of alleles that although shared between populations, have arisen independently in different genetic backgrounds (homoplasy), due to the influence of selection pressures, such as antimalarial drugs . Accurate haplotype reconstruction is a common problem to most genotyping platforms. To facilitate genetic analysis in the presence of multiclonal infections, the common practice is to use the predominant allele detected at each locus to reconstruct “infection haplotypes” or to remove multiclonal infections from analysis (the latter commonly used when performing genotype-phenotype association studies). Anderson and colleagues observed very similar LD estimates between these two datasets types in *P. falciparum* populations from a range of geographic locations, including highly diverse and mutliclonal populations from Africa [19]. Single molecule sequencing approaches such as the Illumina Genome Analyzer platform produce short sequence reads (or read pairs) which are essentially haplotypes. Particularly with increasing read lengths, these datasets should provide critical information to facilitate the imputation of haplotypes from genotyping data. Indeed, whole genome sequencing platforms provide a useful approach to identify features such as signatures of selection and candidate drug resistance or immune evasion loci. With the capacity to genotype a large set of samples and SNPs in parallel, using small quantities of parasite DNA (typical in extracts from filter paper) even in the presence of abundant human DNA, genotyping platforms such as the GoldenGate assay provide an important tool to validate these candidate loci in large sample collections.

In our study, we were able to detect multiclonal infections (as indicated by the presence of heterozygous genotype calls) in clinical samples. In addition to identifying whether a sample carried more than one genetic variant, we were able to describe the extent of diversity within a sample (i.e. how different the parasites within a multiple infection are), as defined by the proportion of heterozygous loci within a sample. Using this method, we observed higher levels of diversity within the clinical African samples than in the clinical PNG samples, consistent with observations from datasets produced on the Illumina Genome Analyzer platform (Manske *et al*., under review). Using artificial mixes of laboratory clones, the 306-SNP assay demonstrated high specificity (high concordance between replicates) and modest sensitivity to detect minor frequency variants (generally sensitive at minor allele frequencies >10%). At this level of sensitivity, using the 306 validated SNPs on 143 clinical and short-term cultured samples from across the globe, we observed global patterns of population genetic diversity and differentiation consistent with previous reports using data generated on other platforms.

Our study also demonstrated a potential utility of the GoldenGate assay in assigning the geographic origin to an infection on the basis of its clustering pattern in PCA and underlying molecular barcode.

As observed in other studies [20], PCA demonstrated distinct clustering of samples by continental boundaries, and moderate clustering by political boundaries. We used this ability to differentiate samples by geographic origin to identify minimal, informative subsets of SNPs, or molecular barcodes, to assign the geographic origin of an infection. A 6-SNP molecular barcode was sufficient to determine whether a sample was from Thailand or Cambodia, and a 4-SNP barcode was sufficient to resolve samples from East and West Africa. Determining whether a sample was from Mali or Burkina Faso was more ambiguous owing to apparent substantial gene flow between these populations. Ongoing whole genome sequencing projects will facilitate identification of informative SNPs for high-resolution molecular barcodes. Using appropriate sampling parameters and SNP sets specific to the population(s) of interest and informed by the latest genome-wide sequencing *Plasmodium* datasets, greater geographic positioning resolution, possibly at the village level, is anticipated. The ability to trace the migratory patterns of parasites has important implications for malaria control, including potential utility in drug resistance surveillance studies and identifying important routes of new infections in populations in the late stages of malaria elimination and at risk of potential resurgence.

In summary, we discuss the challenges of genotyping in *P. falciparum*, and demonstrate high genotyping specificity and moderate sensitivity even on low quantity (2 ng) DNA templates, typical of filter paper extracts. In addition to the high sample and SNP throughput, an important feature of the GoldenGate platform is its custom-design potential. This feature enables researchers to select the specific SNPs which best facilitate their research objective. With ongoing *Plasmodium* whole-genome sequencing efforts and the identification of novel candidate disease and drug resistance loci (Manske *et al*., under review), researches will have a large resource of SNPs from which to select optimal subsets for their specific study design.

## Supporting Information

Figure S1 Distribution of theta values (proxy to genotype call) at 306 SNPs in (left to right) the laboratory clone 3D7 and 2 clinical samples from Burkina Faso and PNG. Values approximating 0 and 1 correspond to homozygous calls while other values represent heterozygous calls.
(TIF)

Figure S2 Principal Components Analysis plots (x axis represents PC1, and y axis PC2) using molecular barcodes to differentiate the 143 samples and assign geographic origins to the infections. Green = Mali Orange = Burkina Faso, Blue = Kenya, Black = Cambodia, Grey = Thailand, Red = Papua New Guinea. Using a 9-SNP molecular barcode, samples can clearly be assigned continental origins (a) Clear sample assignment to East or West Africa is possible using a 4-SNP molecular barcode, although Mali and Burkina Faso cannot be resolved (b). Using a 6-SNP molecular barcode, samples can be assigned to Thailand or Cambodia with moderate confidence (c). The highest resolution of Mali and Burkina Faso is possible with a 13-SNP barcode (additional SNPs do not improve resolution), but assignment to either population remains moderately ambiguous (d).
(TIF)

Table S1 **Sample details.**
(DOCX)

Table S2 **Golden Gate custom assay information.**
(XLSX)

Table S3 **Assessment of assay performance using 319 “reliable” SNPs in the presence of human DNA.** ^a^ Total 250 ng genomic DNA. ^b^ Filtration criteria A: SNPs with genotype concordance between replicates >0.95. ^c^ Filtration criteria B: SNPs with R>0.1 and with genotype concordance between replicates >0.95. ^d^ Mean correlation between replicates.
(DOCX)

Table S4 **Assessment of assay performance using 319 “reliable” SNPs on whole genome amplified (WGA) samples.** ^a^ Filtration criteria A: SNPs with genotype concordance between replicates >0.95. ^b^ Filtration criteria B: SNPs with R>0.1 and with genotype concordance between replicates >0.95.
(DOCX)
