# Supplementary figures and images for: Population Genetic Analysis of Plasmodium falciparum Parasites Using a Customized Illumina GoldenGate Genotyping Assay
Source: PLoS One. 2011 Jun 6;6(6):e20251. doi: 10.1371/journal.pone.0020251 (PMC3108946; doi:10.1371/journal.pone.0020251)

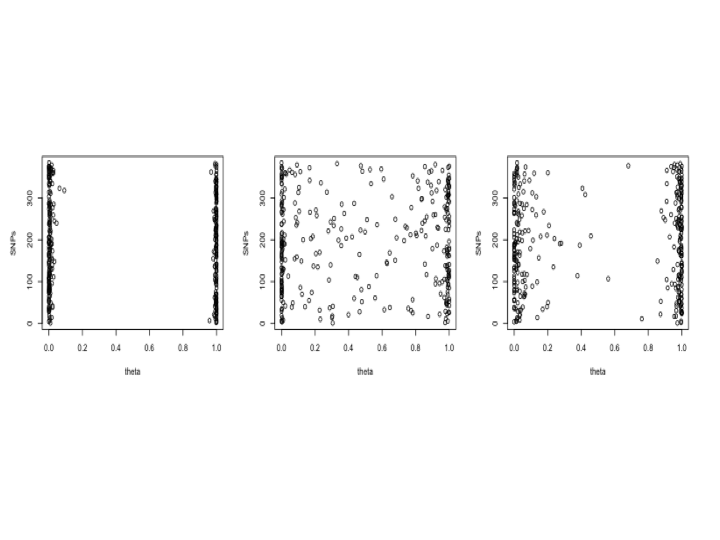

Supplement: Figure S1 — Distribution of theta values (proxy to genotype call) at 306 SNPs in (left to right) the laboratory clone 3D7 and 2 clinical samples from Burkina Faso and PNG. Values approximating 0 and 1 correspond to homozygous calls while other values represent heterozygous calls. (TIF) [file pone.0020251.s001.tif]

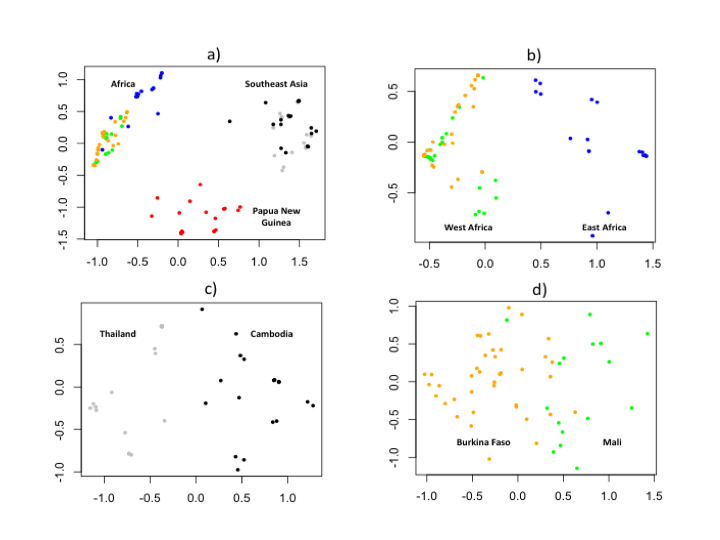

Supplement: Figure S2 — Principal Components Analysis plots (x axis represents PC1, and y axis PC2) using molecular barcodes to differentiate the 143 samples and assign geographic origins to the infections. Green = Mali Orange = Burkina Faso, Blue = Kenya, Black = Cambodia, Grey = Thailand, Red = Papua New Guinea. Using a 9-SNP molecular barcode, samples can clearly be assigned continental origins (a) Clear sample assignment to East or West Africa is possible using a 4-SNP molecular barcode, although Mali and Burkina Faso cannot be resolved (b). Using a 6-SNP molecular barcode, samples can be assigned to Thailand or Cambodia with moderate confidence (c). The highest resolution of Mali and Burkina Faso is possible with a 13-SNP barcode (additional SNPs do not improve resolution), but assignment to either population remains moderately ambiguous (d). (TIF) [file pone.0020251.s002.tif]
