# Supplementary material for: Population Genetic Analysis of Plasmodium falciparum Parasites Using a Customized Illumina GoldenGate Genotyping Assay
Source: PLoS One. 2011 Jun 6;6(6):e20251. doi: 10.1371/journal.pone.0020251 (PMC3108946; doi:10.1371/journal.pone.0020251)
Supplement: Table S1 — Sample details. (DOCX) [file pone.0020251.s003.docx]

**Table S1 - Sample details**

| **Sample ID** | **Geographic Origin** | **Sample size** | **Clinical/culture** |
| --- | --- | --- | --- |
| 3D7 | Unknown (airport malaria) | 1 | Long term culture |
| HB3 | Honduras | 1 | Long term culture |
| IT | Conflicting:Southeast Asia/Brasil | 1 | Long term culture |
| PN | Papua New Guinea | 23 | clinical isolate (non-cultured) |
| PD | Thailand | 19 | Short term culture |
| PH | Cambodia | 22 | Short term culture |
| PC | Kenya | 19 | Short term culture |
| PK | Burkina Faso | 37 | clinical isolate (non-cultured) |
| PM | Mali | 23 | clinical isolate (non-cultured) |
