# Supplementary material for: Population Genetic Analysis of Plasmodium falciparum Parasites Using a Customized Illumina GoldenGate Genotyping Assay
Source: PLoS One. 2011 Jun 6;6(6):e20251. doi: 10.1371/journal.pone.0020251 (PMC3108946; doi:10.1371/journal.pone.0020251)
Supplement: Table S3 — Assessment of assay performance using 319 “reliable” SNPs in the presence of human DNA. a Total 250 ng genomic DNA. b Filtration criteria A: SNPs with genotype concordance between replicates >0.95. c Filtration criteria B: SNPs with R>0.1 and with genotype concordance between replicates >0.95. d Mean correlation between replicates. (DOCX) [file pone.0020251.s005.docx]

**Table S3 - Assessment of assay performance using 319 "reliable" SNPs in the presence of human DNA.**

| **Sample** | **Percent *P. falciparum* DNA ^a^** | **Intensity (R>0.1)** | **Filtration A^b^** | **Filtration B^c^** | **Correlation between replicates^d^** | **Correlation with pure parasite DNA^d^** |
| --- | --- | --- | --- | --- | --- | --- |
|  |  | **nº SNPs** | **nº SNPs** | **nº SNPs** |  |  |
|  |  |  |  |  |  |  |
| 3D7 | 1 | 319 | 319 | 319 | 0.999 | 0.999 |
|  | 0.1 | 317 | 316 | 314 | 0.999 | 0.999 |
|  | 0.01 | 311 | 295 | 292 | 0.993 | 0.992 |
|  | 0.001 | 235 | 271 | 216 | 0.97 | 0.943 |
|  |  |  |  |  |  |  |
| HB3 | 1 | 303 | 310 | 302 | 0.996 | 0.999 |
|  | 0.1 | 302 | 305 | 298 | 0.996 | 0.992 |
|  | 0.01 | 299 | 286 | 278 | 0.988 | 0.98 |
|  | 0.001 | 242 | 256 | 204 | 0.972 | 0.935 |
|  |  |  |  |  |  |  |
| IT | 1 | 304 | 304 | 299 | 0.998 | 0.998 |
|  | 0.1 | 299 | 291 | 285 | 0.985 | 0.985 |
|  | 0.01 | 271 | 259 | 235 | 0.969 | 0.958 |
|  | 0.001 | 174 | 219 | 135 | 0.909 | 0.86 |

^a^ Total 250ng genomic DNA.

^b^ Filtration criteria A: SNPs with genotype concordance between replicates >0.95.

^c^ Filtration criteria B: SNPs with R>0.1 and with genotype concordance between replicates >0.95.

^d^ Mean correlation between replicates.
