# Supplementary material for: Population Genetic Analysis of Plasmodium falciparum Parasites Using a Customized Illumina GoldenGate Genotyping Assay
Source: PLoS One. 2011 Jun 6;6(6):e20251. doi: 10.1371/journal.pone.0020251 (PMC3108946; doi:10.1371/journal.pone.0020251)
Supplement: Table S4 — Assessment of assay performance using 319 “reliable” SNPs on whole genome amplified (WGA) samples. a Filtration criteria A: SNPs with genotype concordance between replicates >0.95. b Filtration criteria B: SNPs with R>0.1 and with genotype concordance between replicates >0.95. (DOCX) [file pone.0020251.s006.docx]

**Table S4 - Assessment of assay performance using 319 "reliable" SNPs on whole genome amplified (WGA) samples.**

| **Sample** | **Sample quantity (ng)** | **Intensity (R>0.1)** | **Filtration A ^a^** | **Filtration B ^b^** | **Correlation between replicates** | **Correlation between gDNA and WGA** |
| --- | --- | --- | --- | --- | --- | --- |
|  |  | **nº SNPs** | **nº SNPs** | **nº SNPs** |  |  |
|  |  |  |  |  |  |  |
| 3D7 WGA | 250 | 317 | 319 | 317 | 0.9999 | 0.9999 |
|  | 25 | 319 | 318 | 318 | 0.9999 | 0.9997 |
|  |  |  |  |  |  |  |
| 3D7:human WGA | 250 | 319 | 318 | 318 | 0.9999 | 0.9998 |
|  | 25 | 319 | 319 | 319 | 0.9999 | 0.9998 |
|  |  |  |  |  |  |  |
| gDNA Mali A | 10 | 313 | 317 | 311 | 0.9998 | - |
| WGA Mali A | 250 | 312 | 310 | 304 | 0.9992 | 0.9989 |
|  | 25 | 312 | 313 | 307 | 0.9973 | 0.9973 |
|  |  |  |  |  |  |  |
| gDNA Mali B | 10 | 316 | 312 | 310 | 0.9989 | - |
| WGA Mali B | 250 | 314 | 249 | 245 | 0.9976 | 0.9961 |
|  | 25 | 315 | 309 | 308 | 0.9930 | 0.9917 |

^a^ Filtration criteria A: SNPs with genotype concordance between replicates >0.95.

^b^ Filtration criteria B: SNPs with R>0.1 and with genotype concordance between replicates >0.95.
